# Design and Development of a Spanish Hearing Test for Speech in Noise (PAHRE)

**DOI:** 10.3390/audiolres13010004

**Published:** 2022-12-30

**Authors:** Marlene Rodríguez-Ferreiro, Montserrat Durán-Bouza, Victoria Marrero-Aguiar

**Affiliations:** 1 Psychology Department, University of A Coruña, 15008 A Coruña, Spain; 2 Spanish Language and General Linguistics Department, Universidad Nacional de Educación a Distancia, UNED, 28040 Madrid, Spain

**Keywords:** speech in noise, hearing assessment, Spanish hearing test

## Abstract

Background: There are few hearing tests in Spanish that assess speech discrimination in noise in the adult population that take into account the Lombard effect. This study presents the design and development of a Spanish hearing test for speech in noise (Prueba Auditiva de Habla en Ruido en Español (PAHRE) in Spanish). The pattern of the Quick Speech in Noise test was followed when drafting sentences with five key words each grouped in lists of six sentences. It was necessary to take into account the differences between English and Spanish. Methods: A total of 61 people (24 men and 37 women) with an average age of 46.9 (range 18–84 years) participated in the study. The work was carried out in two phases. In the first phase, a list of Spanish sentences was drafted and subjected to a familiarity test based on the semantic and syntactic characteristics of the sentences; as a result, a list of sentences was selected for the final test. In the second phase, the selected sentences were recorded with and without the Lombard effect, the equivalence between both lists was analysed, and the test was applied to a first reference population. Results: The results obtained allow us to affirm that it is representative of the Spanish spoken in its variety in peninsular Spain. Conclusions: In addition, these results point to the usefulness of the PAHRE test in assessing speech in noise by maintaining a fixed speech intensity while varying the intensity of the multi-speaker background noise. The incorporation of the Lombard effect in the test shows discrimination differences with the same signal-to-noise ratio compared to the test without the Lombard effect.

## 1. Introduction

Noisy environments undoubtedly lead to a degradation in the intelligibility of speech, and hence make it more difficult to understand the spoken message, especially for older individuals or those with hearing loss. This is so because noise masks part of the speech signal, leading to a loss of acoustic information and thus to an increase in recognition errors [1]. The noises that generate the greatest changes in speech production are those that share spectral components with vocalization, as well as those of greatest intensity, which thus involve a decrease in the signal-to-noise ratio (SNR) [2,3,4,5]. 

SNR loss is not reflected, nor can be predicted, by means of tonal audiometry [6,7,8]. However, there does appear to be a relationship between the degree of hearing loss and the SNR loss. That is, the greater the degree of hearing loss, the greater the SNR loss [9,10,11], especially if this loss affects high-frequency acoustic signals to a greater extent than other frequencies [12]. Because of the difficulties in comprehension by the listener involved in a speech-in-noise situation, especially in older people and/or those with hearing loss, incorporating speech-in-noise tests into hearing tests has been highly recommended [13].

However, the oral communication system has mechanisms to cope with the effects of noise on speech. The most studied of these has been the so-called “Lombard effect”. 

### 1.1. The Lombard Effect

The Lombard effect, first described in 1911 by Etienne Lombard [14], is an unconscious adaptation by the speaker to the noise through a series of changes in speech to improve the intelligibility of the message. Over the last hundred years, the Lombard effect has been studied in countless research studies. In summary, we can say that, even when taking into account differences due to the nature of the speaker, the context, and the environment, most studies agree in pointing out some consequences of the Lombard effect on the signal in interactive situations [2,15,16,17,18,19,20,21]. Table 1 sets out these characteristics.

All these aspects of the Lombard effect typically affect words with greater semantic load the most, with functional words, which tend to have a lower information load, being less affected [5,17]. 

The Lombard effect has been assessed in Spanish, with results similar to those found in other studies of other languages, although in comparison with American English and French there is a tendency in Spanish towards a greater variation of the second formant and of speech intensity [22]. In Junqua [16], significant differences were also found between male and female speakers, with Lombard speech by females being more intelligible. This set of features makes speech produced under the Lombard effect more intelligible than speech produced in silence when presented under equal SNR conditions [3,5,23]. 

As can be deduced from the above information, the Lombard effect goes far beyond a simple increase in signal intensity and it does not only affect the signal-to-noise ratio, but also involves frequency and temporal changes aimed at improving signal perceptibility. A hearing evaluation that aims to reproduce as closely as possible the natural conditions of verbal communication should consider this effect, which occurs naturally and continuously in verbal interactions.

### 1.2. Assessment of Signal-to-Noise Loss Using Speech-in-Noise Tests

Over recent years, tests to assess speech-in-noise and quantify the SNR loss quickly and reliably have gained prominence. Its use has demonstrated many benefits. On the one hand, these tests favour the selection and fitting of hearing aids, providing realistic expectations in terms of their use [6]. On the other hand, they allow for an assessment of the need to add aural rehabilitation to the fitting of hearing aids [24,25,26,27,28]. These tests are also helpful in the post-fitting phases when the effectiveness of hearing aids is assessed and validated [29], as well as in assessing the usefulness of hearing assistive technology such as remote microphones [30,31]. Since then, various speech-in-noise tests have been developed. These tests are differentiated according to the verbal stimulus presented, the SNR used, and approximate application time. Table 2 sets out the main speech-in-noise tests and summarises their primary characteristics.

Research has made it possible to compare the effectiveness of some of the most frequently used tests in the evaluation of signal-to-noise loss. In particular, Duncan and Aarts [39] compared the HINT and QuickSIN tests, noting the advantages of the latter in terms of both usability and sensitivity for subjects with hearing loss. The results of Wilson, McArdle, and Smith [12] indicated that the QuickSIN and WIN tests are more sensitive measures of recognition in background noise than the BKB-SIN and HINT tests. More recently, Sultan et al. [40] reported a higher correlation of the QuickSIN test with the background noise (BN) subscale of the Abbreviated Profile of Hearing Aid Benefit (APHAB) questionnaire [41] as compared to the HINT test, both in normal hearing subjects and in subjects with hearing loss. In a systematic review of 21 studies on the relationship between auditory behavioural measures and hearing aid satisfaction, Davidson et al. [42] conclude that speech-in-noise tests had the highest associations with hearing aid satisfaction, and specifically the QuickSIN was found to be a significant predictor of hearing aid satisfaction.

Although the tests listed are the most frequently used, both in their original English version and in adaptations to other languages, additional tests of this type are still being developed. In some cases, additional tests are developed for use in specific populations, as in the case of AzBio [43], which is used with people with cochlear implants. Other tests have emerged as a consequence of technological advances, greater accessibility by individuals, or lower cost, such as those that allow the initial screening of hearing using mobile phones [44,45,46,47]. 

At present, in Spain, the most frequently used verbal test in audiological assessments is verbal audiometry with lists of words with two syllables [48]. Although this test is very useful for traditional speech audiometry, its effectiveness is reduced when including background noise; in addition, it relies only on single words, not sentences. Isolated monosyllabic or bisyllabic words, recorded and reproduced at uniform intensity levels, are not representative of real-world speech. Sentences are a closer and more realistic representation of everyday speech [35,49,50]. With regards to speech-in-noise tests for the adult population, we have some adapted to Spanish: the Digit Triplet Identification Test [51], the Sentence Matrix Test for Spanish speakers [52], the Spanish adaptation of HINT [53], SPIN adapted for Spanish as Spanish Sentence Lists (LFE in Spanish) [54], and its shortened version [55]. However, these tests have not had a great impact at the clinical level, either because they take a long time to administer, they require the prior training of the patient, or simply because they are not easily accessible by the clinicians. A set of 700 phonetically balanced Spanish sentences is also available called the Sharvard Corpus [56], but verbal material for audiometry in noise requires an added control of variables, as will be discussed below, which prevents the use of these sentences and demands the construction of an ad hoc corpus.

Finally, it is surprising that despite the fact that speakers produce Lombard speech when in background noise to improve both SNR and speech discrimination, few tests take this effect into account when assessing speech intelligibility in noise. Incorporating Lombard speech into a speech-in-noise test would allow for more realistic speech-in-noise discrimination data. In Spanish, the Verbal Noise Hearing Test (Prueba de Audiometría Verbal en Ruido (PAVER) in Spanish) developed by Marrero-Aguiar 57 is itself based on the QuickSIN test. It is the only such test that takes this effect into consideration in the paediatric population. It contains three blocks of sentences, one of which includes the Lombard effect.

Based on all the above, the development of a speech-in-noise test for the adult population in Spanish seems to be useful and indeed necessary. To this end, the QuickSIN test was taken as a model, due to its speed, simplicity of administration, and because of the amount of information it yields. The QuickSIN pattern was maintained in terms of the number of keywords per sentence (five) and the number of sentences in each list (six), but adaptations from English to Spanish involved taking into account the differences between the two languages in terms of lexical quantity and distribution, sentence length, word length, vowel and consonant use, weight of functional and lexical words, sounds, symmetry in orthographic, morphological and syntactic parameters, and similarities in terms of the conceptual organisation underlying the words themselves [58]. The choice of speech materials and background noise will, as Villchur [50] points out, be a compromise between realism and reproducibility. In other words, the aim is to reproduce an everyday speech situation with noise.

Another contribution of this test has been the incorporation of the Lombard effect to compare the results obtained when the speech is presented without this effect (as in the rest of the SIN tests) and when it is a Lombard speech. We assume that the Lombard lists will present a higher score and that it will be maintained with lower SNR values. The results will make it possible to establish the need to incorporate this effect in the SIN tests or to maintain only the recording of the verbal material under the same conditions as has been done so far in the existing speech-in-noise tests.

## 2. Materials and Methods

This paper describes the process followed in the design and development of the lists of sentences that will form part of a Spanish hearing test of speech-in-noise (in Spanish, Prueba Auditiva de Habla en Ruido en Español, PAHRE). In the first phase, a list of sentences was compiled and subjected to a familiarity test based on the type of semantic and syntactic features of the sentences. In the second phase, the sentences were recorded with and without the Lombard effect, after which the equivalence between both lists was analysed and the test was applied.

As in any speech-in-noise test, decisions had to be made regarding the preparation of the verbal material, as well as its recording and playback, taking into account issues such as the characteristics of the speaker, the type of background noise, and the SNR used.

### 2.1. PHASE 1: Familiarity Test

#### 2.1.1. Purpose

The preparation of the lists of six sentences from a total of 240 sentences containing five key words, that are representative of spoken Spanish. 

#### 2.1.2. Participants

The sample consisted of a total of 31 Spanish–Galician bilingual subjects (11 men and 20 women) with a mean age of 46.81 years (range 23–84 years). The educational level of the subjects ranged from basic to master’s degree studies (see Table 3). The inclusion criterion was to be over 18 years of age and not to have cognitive impairments that prevented an understanding the instructions given.

#### 2.1.3. Instruments

The verbal material to be included in the test was taken from a total of 240 sentences, each containing five key Spanish words. The sentences were grouped into lists of six, so that each list contained 30 keywords (a total of 40 lists and 1200 keywords). The complexity of the sentences varied according to semantic and syntactic features. The following criteria were considered in creating this list:

(a) Following CORPES XXI corpus [59], nouns, verbs, adjectives, and adverbs were considered key words, in that these constitute 51.97% of all Spanish word classes. Taking into account the frequency of occurrence of each type of keyword, each list of six sentences with a total of 30 keywords was required to contain fifteen nouns, eight verbs, four adjectives, and three adverbs.
(b) Given that studies vary regarding the number of words that an adult is likely to know (between 34,000 and 50,000 words), and to avoid the influence of factors such as age and socio-educational and cultural level, the lexicon was limited to some of the most familiar and frequently occurring words in the Spanish language. The selection of these was again based on CORPES XXI. Function words, punctuation marks, orthographic marks, fixed word combinations, abbreviations, acronyms, digits, proper names, and Anglicisms were excluded, until 3000 items were reached.
(c) To achieve a phonic and syllabic balance in each list of sentences, we followed the indications set out in the Inventory of Phonemic and Syllabic Frequencies of Spontaneous and Written Spanish (In Spanish, Inventario de Frecuencias Fonémicas y Silábicas del Castellano Espontáneo y Escrito, IFFSCEE) [60].
(d) All sentences were expressed in the declarative form to avoid differences in intonation patterns.
(e) At the syntactic level, the structure of most of the sentences was subject-verb-predicate.
(f) Following Véliz et al. [61], the length and syntactic complexity of the sentences had to allow for their exact and immediate reproduction after their presentation in auditory mode, regardless of the age and working memory of the participants.

The list of 240 sentences was subjected to a familiarity test in which participants were asked to discard those sentences they considered the most complex or unusual based on their lexical and/or syntactic characteristics. In this way, the final list comprised a total of 168 sentences (28 lists of six sentences each).

#### 2.1.4. Procedure

Participants were recruited by means of a text message sent to their phones) describing the purpose of the study to work colleagues and acquaintances. Participation was voluntary, and once the main aim of the study had been explained, participants were asked to sign an informed consent form. This meeting was also used to address and possible queries or doubts that participants might have. 

A document with the 240 sentences was then sent by e-mail to the 31 participants for their assessment, together with brief instructions and a template on which to note observations and unusual sentences. The instructions indicated that they should read each sentence and indicate on the template any that they considered complex and/or unusual, either at a semantic and/or syntactic level, explaining the reasons for their choice. They were asked to complete the assessment within one week.

Once all ratings had been received, the choices made by each of the participants were analysed. The sentences in which there was greater agreement regarding their complexity (four or more people discarded them) were eliminated. In total there were 72 sentences that were removed from the original list, resulting in a list of 168 sentences, these grouped into 28 lists of six sentences. Each list maintained the proportion of keywords indicated above (fifteen nouns, eight verbs, four adjectives, and three adverbs).

#### 2.1.5. Data Analysis

The SPSS statistical package, version 28.0 for Windows, was used to analyse the data obtained in this first phase of the study. A correlation analysis was carried out in order to check the representativeness of the phonemes and syllables included in the Spanish keywords in the lists of the 168 sentences as grouped in 28 lists and presented to the subjects. Through this analysis we were able to verify the relationship between the frequency of the phonemes and syllables of the sentences that were intended to be included in the listening test with those of the IFFSCEE [60], which was used as a reference.

#### 2.1.6. Results

The 28 lists of six sentences resulting from the familiarity test were used to test whether the phonemes and syllables that made up the sentences were representative of the key Spanish words (nouns, verbs, adjectives, and adverbs). A Pearson correlation analysis was carried out to check the relationship between the frequency of occurrence of phonemes and syllables in each of the lists with those of spoken Spanish according to the IFFSCEE [60].

**Figure 1:**
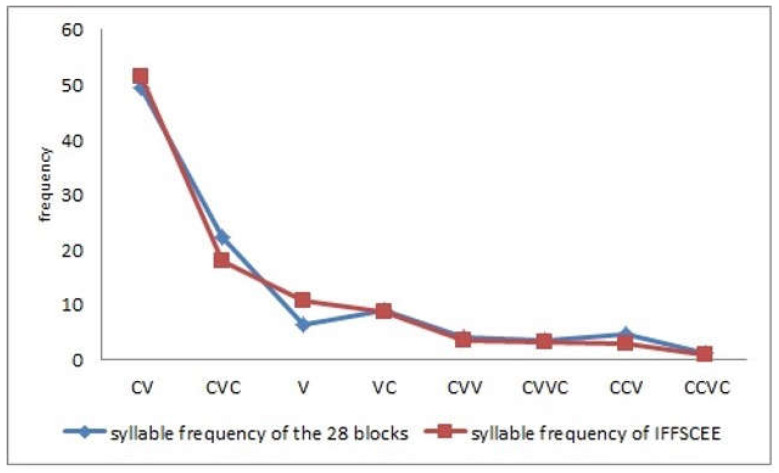
Comparison of syllabic frequencies of the keywords in the 28 lists and in the IFFSCEE.

**Figure 2:**
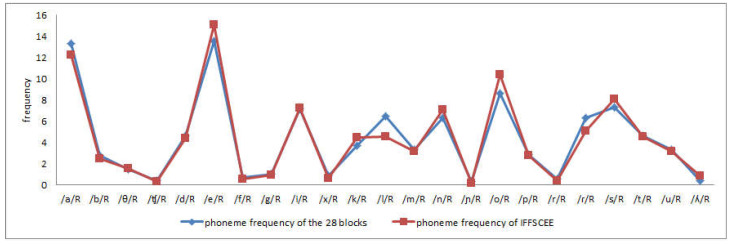
Comparison of phonemic frequencies of the keywords in the 28 lists and in the IFFSCEE.

Taking all the phonemes into account, a statistically significant positive correlation was obtained (*r* = 0.88; *p* ≤ 0.05), with the correlation being higher with respect to syllable frequency (*r* = 0.90; *p* ≤ 0.01). In terms of the frequency of each Spanish phoneme and syllable type, as seen in Table 4, statistically significant positive correlations were obtained for both phonemes and syllables. Figure 1 and Figure 2 show the strong relationship between the syllabic and phonemic frequency of the 28 lists of sentences with the frequencies of those taken as a reference.

### 2.2. PHASE 2: Calculation of Intelligibility and Assessment of the Lombard Effect

#### 2.2.1. Purpose

To assess the level of difficulty and equivalence of the sentences in the normal listening population and to ascertain the incidence of the two types of speech, in silence and under the Lombard effect.

#### 2.2.2. Participants

The sample consisted of a total of 30 Spanish–Galician bilingual subjects (13 men and 17 women) with an average age of 48.37 years (range 18–74 years), with educational levels ranging from elementary schooling to doctorate level (see Table 5). In this case, the inclusion criteria were to be over 18 years of age, to present no cognitive alterations that prevented understanding the instructions given and to present no hearing loss or alterations in speech production. 

To ensure that the participants’ hearing was within the normal hearing range (0–20 dB) and hence that they could participate in the study, all subjects were assessed by means of a battery of hearing tests: otoscopy, pure tone audiometry with TDH 39 headphones, discomfort threshold, and verbal audiometry [48]. Hearing was calculated as the pure tone average of four frequencies (0.5, 1, 2, and 4 kHz) [62,63].

#### 2.2.3. Instruments

Once the 28 lists of sentences from the first phase of this study were obtained, they were recorded. A female speaker was chosen for the recording, as most studies consider female speech to be more intelligible, which is also noted in the recommendations of the International Collegium of Rehabilitative Audiology (ICRA) [64]. Given that the mean fundamental frequency of women’s speech is 200 Hz, with a range from 58 Hz to 274 Hz [65,66,67], a Spanish professional female speaker was sought whose fundamental frequency met these characteristics.

The sentences were recorded in two different listening situations, one with no noise and one with multi-speaker noise presented through headphones to the speaker to generate the Lombard effect. The noise used was multi-speaker noise, since this is more effective in evoking the Lombard effect by sharing spectral components with vocalization.

Since the Lombard effect is more likely to occur at a low SNR [68], different intensity levels were assessed for its presentation. Finally, the SNR was chosen to be low or even negative to avoid generating auditory fatigue in the speaker (78 dB SPL).

The multi-speaker noise was presented to the speaker through insert headphones routed through an application/app, calibrated by the “live listening” functionality to a level of 71, 78 and 80 dB SPL in order to generate the Lombard effect. Recorded speech with a noise of 78 dB SPL was used since the Lombard effect was better represented with this intensity without causing excessive, exaggerated, and unnatural hyper-articulation. The four-person, multi-speaker noise (three females and one male) originally used in Marrero-Aguiar et al. [69] was employed, with the authors’ permission, in that it allows both energetic and informational masking to be generated. The speech was recorded with a Gefell M930 cardioid condenser microphone, about 30 cm away from the speaker and pre-amplified by JZ Track, digitised at 44.1 kHz and 16 bits through the Avid HD-Omni interface with Avid Protocols software. 

In order to vary the SNR in each sentence of the list, a fixed intensity was maintained for the sentences while the intensity of the noise was varied following the QuickSIN model. The noise used was the same multi-speaker noise used for the recordings. Since the purpose of a SIN test is the SNR loss evaluation of individuals with hearing difficulty, the recorded material was applied to five people with hearing loss to determine the choice between the SNR values to be used. The five participants had a bilateral moderate sensorineural hearing loss. The goal was to achieve maximum speech discrimination with the highest SNR and a progressive decrease to zero discrimination at the lowest SNR. The results here showed that the possibility of obtaining a score of 100% in the first sentence became 0% in the last sentence using the SNR +15, +9, +6, +3, 0, and −3 dB for the lists without the Lombard effect. In the case of the lists with the Lombard effect, these same results were obtained with the SNR +12, +6, +3, 0, −3, and −6 dB. Although the results varied between the standard list and with the Lombard effect list, it was decided to use the SNR from +12 to −6 dB, otherwise 0% would not be attained in the list with the Lombard effect. The latency between each of the sentences was eight seconds, enough time for the subject to repeat the sentence comfortably, taking as a reference the QuickSIN test as well as the duration of the recorded sentences in this test. 

#### 2.2.4. Procedure

Participants were selected by sending an SMS explaining the purpose of the study to work colleagues and acquaintances. Participation was voluntary, and once the main aims of the study to be carried had been explained, participants were asked to sign an informed consent form. 

After being assessed through a battery of hearing tests to ensure normal hearing values, they were presented with the 28 lists of sentences without the Lombard effect and instructed to repeat each of the sentences or the parts that they perceived. The lists were presented in random order and binaurally through TDH39 headphones at an intensity of 60 dB HL considering previous studies with speech-in-noise tests in normal-hearing subjects [37,70]. An Equinox 2.0 clinical audiometer was used. The tests were carried out in an acoustically conditioned room, and all the pauses that were deemed convenient were granted either at the request of the subject or at the discretion of the assessor, in order to minimise the effects of fatigue and/or lack of attention. There was no maximum number of breaks allotted, although none of the participants had exceeded three breaks.

When applying the test, each correctly repeated key word was scored with 1 point. Hence, the maximum score for each list was 30 points. Subjects’ verbal productions were recorded to collect data on the keywords in each sentence that were repeated correctly, and thus totals for each list.

After a minimum of two weeks and a maximum of four weeks, the same subjects were presented with the lists with Lombard effect sentences under the same conditions. This period between the presentation of the two lists was intended to avoid possible auditory memory and learning effects. However, this period of time may not be sufficient to eliminate the memory of lexical items. It is assumed that items not perceived in the sentences without the Lombard effect will be perceived as new in the presentation of the Lombard sentences. For this reason, the lists of sentences were presented in this order. The instructions were also the same as for the presentation of the sentences without the Lombard effect, that is, subjects were asked to repeat either the sentence or the parts of it that they had perceived. Once again, pauses were taken between the presentation of the 28 lists of sentences to avoid fatigue and lack of attention. 

As in the lists without the Lombard effect, each correctly repeated key word was scored with 1 point, and subjects’ verbal productions were recorded to collect data on the keywords in each sentence that were repeated correctly, and thus totals for each list.

#### 2.2.5. Data Analysis

The SPSS statistical package (version 28.0) for Windows was used to analyse the data obtained in this phase of the study. Two-factor analyses were performed to test the main components of the 28 lists in both conditions (with and without the Lombard effect). These analyses made it possible to assess the inclusion of the total number of lists in the test, and to judge the scope for reducing them without losing reliability. Cronbach’s alpha coefficient was used to calculate reliability.

Finally, once the normality of the data had been confirmed, a T-test for related samples was used to test the possible existence of differences with respect to the total score obtained by the participants in both lists. In addition, considering the RSR levels, differences between the two lists with respect to the decrease in speech intelligibility were tested by applying non-parametric tests.

#### 2.2.6. Results

The equivalence between the lists of sentences presented without the Lombard effect and those presented with the Lombard effect was analysed. For this purpose, an exploratory factor analysis was carried out with each of the lists. For the block without the Lombard effect, a statistically significant Bartlett’s test was obtained (χ2 (378) = 632.15, *p* ≤ 0.001, and Kaiser-Meyer-Olkin (KMO) = 0.36), allowing for factor clustering. A main component analysis yielded eight main components with eigenvalues > 1; that is, the lists of sentences were classified within eight factors. Factor 1 accounted for 36.34% of the variance, and factor 2 accounted for 10.27%. Together, these two factors explained 46.61% of the total variance. Factor 1 contained 21 of the 28 lists of sentences, while factor 2 contained only 4 of the 28 lists.

A statistically significant Bartlett’s test (χ2 (378) = 613.44, *p* ≤ 0.001, and KMO = 0.15) was also obtained for the block with the Lombard effect, allowing for factor clustering. A main component analysis yielded nine main components with eigenvalues > 1; that is, in this case, the lists of sentences were classified in nine factors. Factor 1 explained 27.86% of the variance, and factor 2 explained 9.63%. Together, these two factors explained 37.49% of the total variance. Factor 1 contained 16 of the 28 lists of sentences, while factor 2 contained 3 of the 28 lists.

Cronbach’s alpha coefficient was calculated in order to test the reliability of the 21 lists that were part of factor 1 in the lists without the Lombard effect and of the 16 lists of factor 1 in the block with the Lombard effect. In both cases, high reliability was obtained (α = 0.86 and α = 0.89, respectively). In order to equalise the number of lists that would be part of the test, the last 5 of the lists without the Lombard effect factor 1 lists were discarded, thus adjusting to the 16 lists with the Lombard effect factor 1. 

Thus, the resulting verbal material comprised two blocks of 16 lists of sentences each, one of the blocks without the Lombard effect and the other with the Lombard effect. 

Giving a score of one point for each correctly repeated keyword allowed for a maximum score for each sentence list of 30 points, and the maximum score that could be attained was thus 480 after scoring the 16 lists of the block without the Lombard effect and the same maximum for the 16 lists of the block with the Lombard effect.

**Figure 3:**
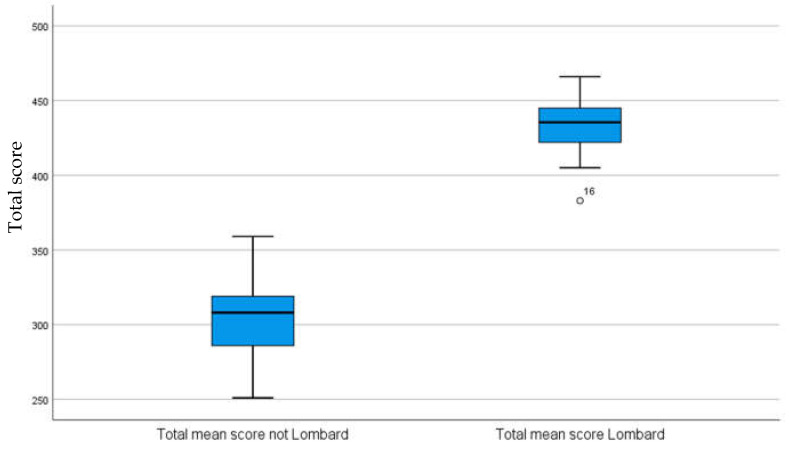
Average total scores for the 16 sentence blocks with and without the Lombard effect.

Once both blocks had been applied to the 30 participants, we looked at whether there were differences in the total score obtained in each of the lists. First, the normality of the data was analysed using the Shapiro–Wilk test and the results here showed that the scores had a normal distribution for both the block without the Lombard effect (W (30) = 0.97, *p* = 0.64) and the block with the Lombard effect (W (30) = 0.98, *p* = 0.70). Therefore, a T-test for related samples was applied, obtaining statistically significant differences with respect to the scores obtained by the subjects in both blocks of lists (t (29) = −54.20, *p* ≤ 0.001), the mean scores of the block with the Lombard effect (see Figure 3) being higher.

In conclusion, participants performed better when sentence lists were presented with the Lombard effect.

**Figure 4:**
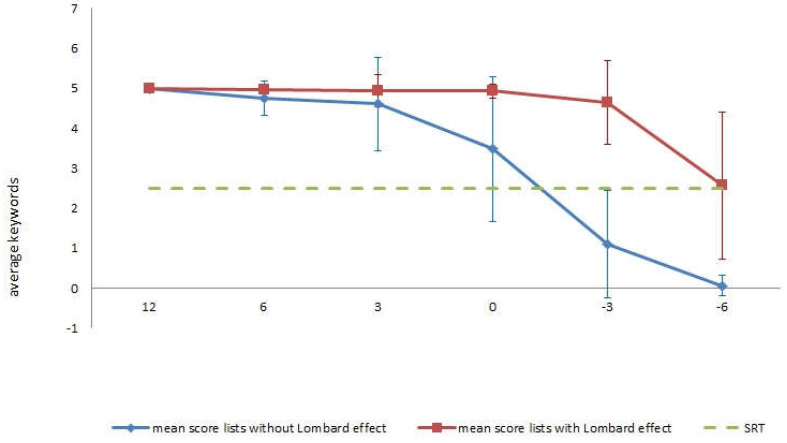
Comparison of speech discrimination as a function of different SNR levels for lists with and without the Lombard effect.

Finally, the scores obtained by the participants were analysed according to the different levels of SNR in the presentation of the sentence lists. As indicated above, the 30 subjects did not have any hearing loss, and although they were of different ages (range 18–74 years), all of them were able to repeat the five key words of the sentences without the Lombard effect for the positive SNRs. However, speech discrimination from the SNR of 0 dB started to decrease, with no repetition of any words at SNR of −6 dB. In the case of the lists with the Lombard effect, speech intelligibility was maintained up to a SNR of −3 dB, and participants were even able to repeat around 3 words with a SNR of −6 dB. Figure 4 shows these results graphically. The dashed line shows the SRT so it can be seen that there is a SRT difference greater than 4dB between the non-Lombard lists and the Lombard lists. These results are in line with those obtained previously when the total scores achieved in both lists were compared.

Since the Shapiro–Wilk test showed that the scores obtained, taking into account SNR levels, did not meet the criteria of normality, the Wilcoxon signed-rank test was applied to analyse the differences between the two blocks with respect to the number of repeated words. The results obtained are shown in Table 6.

These results confirm that participants scored higher on the list with the Lombard effect on both positive and negative SNR levels. An examination of the ranges revealed that at the +12 dB levels, 23 of 30 subjects scored similarly on both lists and only five scored higher on the list with the Lombard effect. At the +6 and +3 dB levels, while there was little variability in the scores obtained in the two lists (as shown in Figure 4), 26 subjects obtained higher scores in the list with the Lombard effect. As already indicated, the differences are clear at the negative SNR levels, with all 30 subjects having higher scores on the list with the Lombard effect.

Again, the results indicate the Lombard speech benefits on word recognition in noise.

The data obtained after the analysis of the results allowed us to establish a hearing test in which a list of sentences from the block without the Lombard effect and a list of sentences from the block with the Lombard effect were randomly paired to form 16 pairs of lists.

Three blocks were elaborated to be used as training by some patients if necessary. These blocks are composed from the three discarded Lombard lists of the factor two and three of the five discarded non-Lombard lists of the factor 1. The application time for each pair of sentence lists in the binaural condition was three minutes.

## 3. Discussion

As already indicated, there are currently very few Spanish-adapted tests of speech-in-noise for the adult population (Digit Triplet Identification, Sentence Matrix Test for Spanish speakers, HINT adapted to Spanish, SPIN adapted to Spanish as Spanish Sentence Lists (LFE), and its reduced version), and they offer little scope in terms of having an impact at the clinical level, sometimes because they involve long application times. The Spanish hearing test for speech-in-noise (PAHRE) aims to overcome some of these previous drawbacks. It is short, since its application in binaural conditions does not exceed three minutes, easy to administer through any clinical audiometer without the need for prior preparation or complex explanations for the patient, but with high reliability when assessing speech-in-noise.

The results obtained from the analysis of the material described here have allow us to confirm that this material is indeed representative of spoken Spanish both at a semantic, syllabic, and phonetic level. Therefore, it can be included as a speech-in noise test to complete the hearing tests that are carried out in the clinic in Spain. We found that both the length of the sentences and their syntactic complexity are such that their exact and immediate reproduction is possible following their presentation in auditory mode, regardless of the age of subjects. These findings reflect the criteria set out in Véliz et al. [61], which were taken into account when designing the hearing test.

Although the QuickSIN speech-in-noise test [37] has been taken as a reference for the development of our Spanish auditory test, a series of new aspects have been introduced with respect to the original test. First, the Lombard effect was incorporated, given that it is characteristic of vocal production in situations with background noise. It was considered essential to incorporate this effect into a speech-in-noise test as it more realistically reflects vocal production and, thus, characteristic speech discrimination in a noisy environment. However, we wanted to also include spoken speech in silence to make the test equal to the rest of the speech-in-noise tests. Thus, in PAHRE, two blocks of Spanish keyword sentences were used, one without the Lombard effect and the other with the Lombard effect. This test complements the one developed in Spanish by Marrero-Aguiar [57] for the paediatric population, given the scarcity of hearing tests for the adult population that incorporate the Lombard effect. 

The spoken-in-silence block (without the Lombard effect) provide similar results to those obtained with most of the speech-in-noise tests, which allows them to be compared when presented in the same conditions. On the other hand, the block with the Lombard effect allows to evaluate speech discrimination in conditions more representative of a noisy environment by incorporating the vocal characteristics that are generated in a speaker in these conditions. The results obtained in both blocks allow comparisons to be made regarding speech discrimination with the same SNR values.

As expected, and in line with previous studies [3,5,23,57], it was confirmed that the lists recorded under the Lombard effect are more intelligible than the lists recorded in silence, maintaining greater discrimination as SNR decreases and greater intelligibility when presented in the same SNR conditions.

Incorporating the list of sentences with the Lombard effect slightly increased the time of the application of the test with respect to the QuickSIN, yet it still does not exceed two minutes in the binaural condition, and in this sense, we consider that the cost-benefit ratio is positive, given the extra information that it provides. 

Another difference of PAHRE with respect to QuickSIN was not incorporating band-pass filtered lists in any frequency range as the purpose of the test is to administer the test to people with different degrees of hearing loss. 

Finally, in contrast to QuickSIN, in PAHRE variations have been made in the SNR, starting from a positive value of less than +25 dB, in addition to decreasing the SNR until reaching negative values in SNR steps of less than 5 dB (except between the first and second sentence). The different SNR values had to allow total discrimination in the first sentence and null discrimination in the last sentence, obtaining progressively lower results as the SNR decreased. It was decided to start the test with SNR +12 dB as it was verified that the participants could repeat all the words with this SNR both in the Lombard and non-Lombard conditions. Therefore, it was unnecessary to include higher SNR values as they did not provide information of interest. The decision regarding the descent of the steps was based on the results obtained. Although with the SNR +15, +9, +6, +3, 0, and −3 dB, the purpose established in the non-Lombard block was achieved, and in the case of the Lombard block null discrimination was not achieved with SNR −3 dB. Since one of the points of interest in the elaboration of this test was the incorporation of the Lombard effect, it was decided to use the SNR +12, +6, +3, 0, −3, and −6 dB to achieve the marked purpose as well in the Lombard block although this decision implied zero discrimination in the last two consecutive steps of the non-Lombard lists. This difference with respect to discrimination based on SNR between the two lists is due to the characteristics of Lombard speech (increased intensity, greater emphasis on high frequencies, reduced speech speed, and more pronounced articulation) that imply large benefits for speech discrimination in noise.

The aim here was to ensure that the person being tested starts the test successfully, and also to obtain more precise information by using 3 dB steps (smaller steps were discarded because of their impact on the time taken to carry out the test, with consequently greater effort and fatigue by the person being tested). These aspects are considered essential because they allow the improvement of the selection and fitting of the hearing aids based on its advanced features to improve SNR, such as directionality and digital noise reduction [25,27,28,71]. Prospective studies of this test will be necessary to assess the speech discrimination loss as the SNR decreases for different age groups in the normal hearing people. Similarly, it will also be necessary to obtain data on the speech reception threshold (SRT) in noise for these same age groups. In this way, this information will allow assessment of the SNR loss in the people with hearing loss and, therefore, the degrees of speaking in noise difficulty as established by the QuickSIN test (mild, moderate, or severe). Based on these results, patient-centred clinical decisions can be made regarding the advanced features of the hearing aid, the use of hearing assistive technology taking as an example the recent study by Davidson et al. [71], or even the need for auditory training.

The results obtained from future studies will present information for both conditions (no Lombard and Lombard). We assume that both the speech discrimination curves as a function of SNR as well as SRT will be better in the Lombard lists for any age group. These future studies will establish the need to maintain both lists in the test due to the information obtained with them and the usefulness of this information for auditory rehabilitation or, on the contrary, to limit the test to one of these two lists.

Limitations of the current study include the decision not to take into account influential variables in the loss of signal-to-noise ratio such as age, speech intelligibility index, and cognitive processing (cf. Humes [72]). Such variables might indeed explain differences in the results obtained when comparing the data on both lists. In the future, and with a larger sample of people of different ages and hearing conditions, these variables will be considered, so that reference curves can be obtained for clinical use.

Although the duration of this test is short and the level of demand required by the patient is limited, we cannot guarantee that it will not cause fatigue since no type of measure has been used to assess it.

Finally, although this data has not been studied or analysed, we believe that this test could be used by non-Spanish-speaking clinicians who work with Spanish-speaking patients with the help of an interpreter. The simplicity of the test and the added help of the response format in which the key words to be repeated by the patient are highlighted, facilitate the administration and evaluation of the test by the interpreter. For this reason, we consider that prior informative training for the interpreter would be sufficient without the need for detailed training. The results for each SNR as well as the SNR loss could be obtained directly and communicated to the patient.

## 4. Conclusions

This study describes the design and development of PAHRE, a speech-in-noise test for Spanish-speaking adults using the QuickSIN test [37] as a model, given its proven sensitivity and effectiveness at the clinical level. The results confirmed the usefulness of the PAHRE test in assessing speech-in-noise, maintaining a fixed speech intensity while varying the intensity of the background multi-speaker noise. This test provides professionals with information about the effect of noise on speech discrimination easily and quickly, without causing fatigue to the person being assessed due to the short duration of the test (3 min). In addition, the assessment can be carried out using a clinical audiometer. It also provides information about speech discrimination in noise in the presence or absence of the Lombard effect. Although we still need prospective studies, the information obtained here will be useful for people with hearing loss to effectively address their difficulties in understanding speech-in-noise and will allow individualised hearing therapy to be proposed and adjusted to each person’s needs and expectations.

## Figures and Tables

**Table 1:** Characteristics of the Lombard Effect in Interactive Situations.

- Signal energy enhancement - Tilt of the speech spectrum that emphasises higher frequencies, where background noise has less energy - Increased vowel duration and intensity, leading to an overall reduction in the speed of the spoken word - Increased syllable duration - Increase in fundamental frequency (F0) - Increase in the frequency of the first formant (F1) and the second formant (F2) - More pronounced facial movements (e.g., lips and jaw)

**Table 2:** Main tests for signal-to-noise loss assessment.

Speech in Noise Tests	Stimulus Presentation	Signal-to-Noise Ratio	Approximate Application Time
Speech Perception in Noise test (SPIN) [32]	Multi-speaker background noise	Fixed SNR of +8 dB	
Matrix test [33]	Noise with spectral speech matching	Variable speech intensity and fixed noise intensity with variable SNR	4–6 min with 8 min of pre-training
Digit in Noise test [34]	Multi-speaker background noise	Different versions	3 min
Hearing in Noise test (HINT) [35]	Male speaker with spectrally matched noise weighted speech	Variable speech intensity in 2 dB steps and fixed noise intensity	5–10 min
Words-in-Noise test (WIN) [36]	Female speaker with multi-speaker noise	Variable speech intensity and fixed noise intensity in 4 dB steps with SNR from +24 to 0 dB	
Quick Speech-in-Noise (QuickSIN) [37]	Female speaker with multi-speaker noise	Fixed speech intensity and variable noise intensity in 5 dB steps with SNR from +25 to 0 dB	2–3 min
Bamford-Kowal-Bench Speech-in-Noise (BKB-SIN) [38]	Male speaker with multi-speaker noise	Fixed speech intensity and variable noise intensity in 3 dB steps with SNR of +21 to 0/−6 dB	6 min

**Table 3:** Socio demographic characteristics of group 1.

		N	Average Age
**Gender**	Male	11	46.91
	Female	20	46.75
	Total	31	46.81
**Level of education**	Basic Education	3	
	Intermediate Vocational Training	3	
	Advanced Vocational Training	6	
	Baccalaureate	2	
	University Studies	14	
	Master’s Degrees Courses	3	
	Total	31	

**Table 4:** Results of Pearson’s correlation analysis of the frequency of occurrence of phonemes and syllables in the drafted lists and those of the IFFSCEE.

List Sentences	r Phonemes	r Syllables
**1**	0.97 **	0.98 **
**2**	0.91 **	0.99 **
**3**	0.91 **	0.96 **
**4**	0.91 **	0.98 **
**5**	0.95 **	0.98 **
**6**	0.92 **	0.97 **
**7**	0.96 **	0.98 **
**8**	0.91 **	0.98 **
**9**	0.96 **	0.99 **
**10**	0.92 **	0.99 **
**11**	0.97 **	0.99 **
**12**	0.94 **	0.96 **
**13**	0.92 **	0.99 **
**14**	0.92 **	0.98 **
**15**	0.96 **	0.91 **
**16**	0.97 **	0.95 **
**17**	0.96 **	0.96 **
**18**	0.93 **	0.97 **
**19**	0.88 **	0.98 **
**20**	0.94 **	0.98 **
**21**	0.94 **	0.99 **
**22**	0.93 **	0.99 **
**23**	0.93 **	0.99 **
**24**	0.90 **	0.98 **
**25**	0.97 **	0.98 **
**26**	0.93 **	0.95 **
**27**	0.95 **	0.95 **
**28**	0.96 **	0.97 **

** *p* ≤ 0.01

**Table 5:** Socio-demographic characteristics of group 2.

		N	Average Age
**Gender**	Male	13	48.23
	Female	17	48.47
	Total	30	48.37
**Level of Education**	Basic Education	5	
	Intermediate Vocational Training	4	
	Advanced Vocational Training	7	
	Baccalaureate	1	
	University Studies	11	
	Master’s Degrees Courses	1	
	PhD Degree	1	
	Total	30	

**Table 6:** Wilcoxon signed-rank test results for different SNR levels.

RSR Levels (dB)	*Z*
L+12 − NL+12	−1.26
L+6 − NL+6	−4.47 **
L+3 − NL+3	−4.52 **
L0 − NL0	−4.78 **
L−3 − NL−3	−4.79 **
L−6 − NL−6	−4.78 **

** *p* ≤ 0.001; L = *Lombard* block; NL = non-Lombard block.

## Data Availability

https://figshare.com/s/5fa59e0a628392cfcfb8 (accessed on 14 November 2022).
